# Supplementary material for: FRET-enhanced photostability allows improved single-molecule tracking of proteins and protein complexes in live mammalian cells
Source: Nat Commun. 2018 Jun 28;9:2520. doi: 10.1038/s41467-018-04486-0 (PMC6023872; doi:10.1038/s41467-018-04486-0)
Supplement: Supplementary file 1 — Supplementary Information [file 41467_2018_4486_MOESM1_ESM.pdf]

FRET-enhanced photostability allows improved single-molecule tracking of proteins and protein complexes in live mammalian cells

Basu and Needham et al.

SUPPLEMENTARY INFORMATION

SUPPLEMENTARY INFORMATION

Supplementary Tables

Supplementary Table 1 | Microscope and experiment details.

Supplementary Table 2 | Modeling the FLIM data using a Bayesian information criterion (BIC).

Supplementary Table 3 | Single-molecule characterization of the mEos3.2-JF646 FRET pair with and without Trolox.

Supplementary Figures

Supplementary Figure 1 | Properties of the mEos3.2 or PA-JF_{549} donor PM fluorophores and the JF_{646} acceptor dye.

Supplementary Figure 2 | FRET and photo-conversion confirmed using bulk fluorescence spectra of mEos3.2-HaloTag constructs.

Supplementary Figure 3 | Heterogeneity in FRET efficiency observed in mEos3.2-JF_{646} single-molecule traces.

Supplementary Figure 4 | Localization precisions calculated for mammalian cell single-molecule imaging

Supplementary Figure 5 | mEos3.2-JF_{646} allows tracking of single CENP-A protein complexes.

Supplementary Figure 6 | Concept of the PA-JF_{549}-JF_{646} FRET *in vitro* pair.

Supplementary Figure 7 | Single-molecule characterization of the PA-JF_{549}-JF_{646} FRET pair.

Supplementary Figure 8 | Several FRET states determined for PA-JF_{549}-tagged histone H2B molecules in the presence of JF_{646}-tagged H2B.

Supplementary Table 1 | Microscope and experiment details.

Figure	Fluorophore	Condition	Buffer	Objective	Total Magnification	Pixel size (nm)	Excitation wavelength (nm)	Power density $kWcm^{-2}$	Imaging mode	Exposure time (ms)	Emission filter	Camera	EM gain	Camera gain	No. of frames
1d,2,Supp Fig 4,5	mEos3.2/mEos3.2-JF_{646}	*In vitro*	PBS, 2 mM Trolox	Olympus 60x NA 1.49 TIRF	135x	109	561	0.4	TIRF	500	BLP-561R and FF01-587/35)	Photometrics Evolve 512	250	6.8	500
Supp Fig 8,9	PA-JF_{549}/ PA-JF_{549}-JF_{646}	*In vitro*	PBS, 1mM DTT, 2 mM Trolox	Olympus 100x NA 1.40 TIRF	100x	160	561	0.4	TIRF	500	BLP-561R and FF01-587/35)	Photometrics Evolve 512	250	6.3	500
3, Supp Fig 6	mEos3.2/mEos3.2-JF_{646}	Live	GMEM/mLIF, 5 mM Trolox	Olympus 60x NA 1.49 TIRF	135x	109	561	0.74	Oblique-angle	500	BLP-561R and FF01-587/35)	Photometrics Evolve 512	250	6.8	10000
4, Supp Fig 6,7	mEos3.2/mEos3.2-JF_{646}	Fixed	PBS, 5 mM Trolox	Olympus 100x NA 1.40 TIRF	100x	160	561	1.34	Oblique-angle	500	BLP-561R and FF01-587/35)	Photometrics Evolve 512 Delta	250	6.3	5000
5, Supp Fig 6	PA-JF_{549}/ PA-JF_{549}-JF_{646}	Live	GMEM/mLIF, 5 mM Trolox	Olympus 100x NA 1.40 TIRF	100x	160	561	1.34	Oblique-angle	500	BLP-561R and FF01-587/35)	Photometrics Evolve 512 Delta	250	6.3	5000

Supplementary Table 2 | Modeling the FLIM data using a Bayesian information criterion (BIC).

Protein	Relative Likelihood (BIC) of $I_{Bkgd} + \sum_i a_i \exp(-t/T_i)$			Values for $I_{Bkgd} + a_1\exp(-t/T_1) + a_2\exp(-t/T_2)$				
	i = 1	i = 2	i = 3	$a_1/(a_1+a_2)$	$a_2/(a_1+a_2)$	T_1(ns)	T_2(ns)	T_{av}(ns)
mEos3.2	0.000016	1	0.0035	0.93 ± 0.02	0.07 ± 0.02	3.80 ± 0.07	0.98 ± 0.09	3.61 ± 0.01
mEos3.2-JF_{646}	2.9×10^{-91}	1	0.0098	0.71 ± 0.04	0.29 ± 0.04	2.38 ± 0.10	0.49 ± 0.04	1.83 ± 0.13
mEos3.2 (Trolox)	2.2×10^{-12}	1	0.0028	0.93 ± 0.01	0.08 ± 0.01	3.71 ± 0.14	0.68 ± 0.19	3.48 ± 0.13
mEos3.2-JF_{646} (Trolox)	1.4×10^{-115}	1	0.76	0.72 ± 0.03	0.28 ± 0.03	2.25 ± 0.01	0.47 ± 0.02	1.75 ± 0.05
PA-JF_{549}	2.3×10^{-47}	1	0.0031	0.90 ± 0.01	0.10 ± 0.01	3.17 ± 0.05	0.55 ± 0.04	2.90 ± 0.04
PA-JF_{549}-JF_{646}	1.5×10^{-22}	1	0.0054	0.76 ± 0.05	0.24 ± 0.05	2.6 ± 0.3	0.44 ± 0.06	2.1 ± 0.4

Tail-fit analysis of the raw data was carried out within the time window of 1.5 <= τ <= 7 ns. [This time window is not affected by the back reflection of the white light laser in the Leica instrument that appears at ~7.5 ns when the laser is run at 80 MHz – this results in the small peak seen in the time normalized data in **Fig. 1c**] The experimental FLIM data were fitted using a Bayesian Information Criterion (BIC) (see **Methods**). This analysis confirms that the most likely model for all the data is two components. The relative amplitude of the two components are $a_1/\Sigma a_i$ and $a_2/\Sigma a_i$, and the lifetimes are τ_1 and τ_2. The weighted average lifetime is given as τ_{av}. The decrease in fluorescence lifetime for mEos3.2 and PA-JF_{549} in the presence of the JF_{646} dye can be clearly observed.

Supplementary Table 3 | Single-molecule characterization of the mEos3.2-JF$_{646}$ FRET pair with and without Trolox.

	No Trolox		Trolox	
	mEos3.2	mEos3.2-HaloJF$_{646}$	mEos3.2	mEos3.2-HaloJF$_{646}$
Emitted photons (10^4)/frame	0.35 ± 0.04	0.40 ± 0.02	0.34 ± 0.01	0.208 ± 0.003
Total emitted photons (10^4)	2.4 ± 0.4	4.1 ± 0.5	2.0 ± 0.1	9.4 ± 0.4
Emitted photons (10^4)/switching event	1.1 ± 0.2	2.6 ± 0.3	1.26 ± 0.09	5.4 ± 0.2
Total on-state time (s)	3.4 ± 0.3	5.2 ± 0.6	2.9 ± 0.2	22.7 ± 0.9
On-state time (s)	1.6 ± 0.1	3.2 ± 0.4	1.8 ± 0.1	13.0 ± 0.5
Switching event number	2.2 ± 0.1	1.62 ± 0.06	1.56 ± 0.04	1.74 ± 0.03
$K_{on} = 1/\tau_{on}$	0.13 ± 0.01	0.086 ± 0.008	0.083 ± 0.006	0.043 ± 0.001
$K_{off} = 1/\tau_{off}$	0.64 ± 0.06	0.31 ± 0.04	0.55 ± 0.03	0.077 ± 0.003
K_{on}/ K_{off}	0.20 ± 0.02	0.28 ± 0.04	0.15 ± 0.01	0.56 ± 0.03

The mean and standard deviations of parameters were extracted as described in **Fig. 2a** and the **Methods**.

a

Fluorophore	Quantum yield
mEos3.2	0.55
PS-mOrange	0.28
PS-CFP2	0.23
PA-mCherry	0.46
PA-TagRFP	0.38
PA-JF_{549}	0.88

b

Fluorophore	Permeability
JF_{646}	Good
Atto655	Good
Alexa Fluor 647	Poor
PA-JF_{549}	Good

c

Fluorophore	Extinction coefficient (M^{-1} cm^{-1})
JF_{646}	152 000
Atto655	125 000
Alexa Fluor 647	239 000

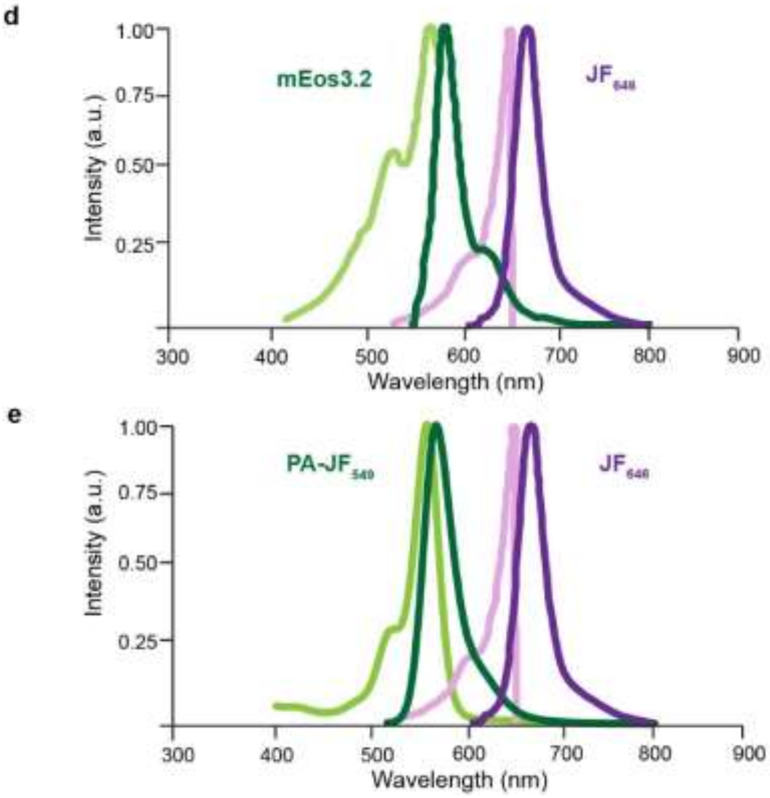

Supplementary Figure 1 | Properties of the mEos3.2 or PA-JF_{549} donor PM fluorophores and the JF_{646} acceptor dye. a) Comparison of quantum yield of potential photo-modulatable donor fluorophores. **b)** Comparison of membrane permeability of organic dyes. **c)** Comparison of extinction coefficients of potential photo-stable acceptor dyes. **d)** Spectral overlap between the mEos3.2 emission and JF_{646} excitation spectra. **e)** Spectral overlap between the PA-JF_{549} emission and JF_{646} excitation spectra.

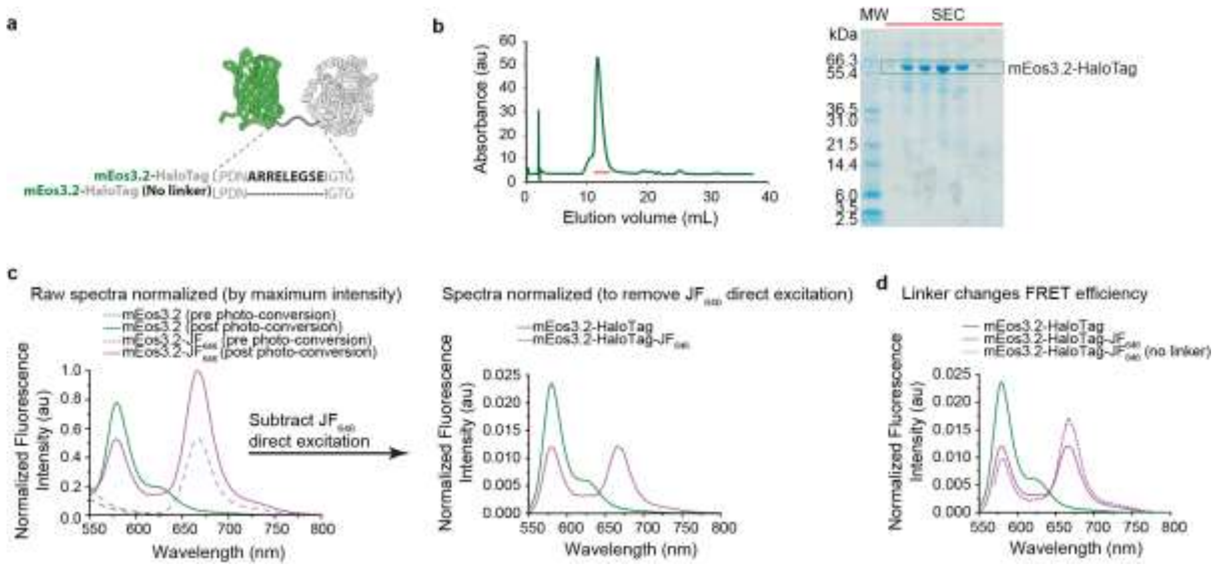

Supplementary Figure 2 | FRET and photo-conversion were confirmed using bulk fluorescence spectra of mEos3.2-HaloTag constructs with and without a linker. a) Sequences of the linker region of the two constructs. **b)** Purification of the mEos3.2-HaloTag fusion protein. **c)** Bulk fluorescence spectra for mEos3.2-HaloTag with and without JF_{646}. The spectra were normalized by maximum intensity using the emission spectra of the pre-converted proteins (left) and, additionally, to remove the effects of direct excitation of the JF_{646} (right). **d)** Comparison of bulk fluorescence spectra for mEos3.2-HaloTag with and without JF_{646}, and with and without the linker.

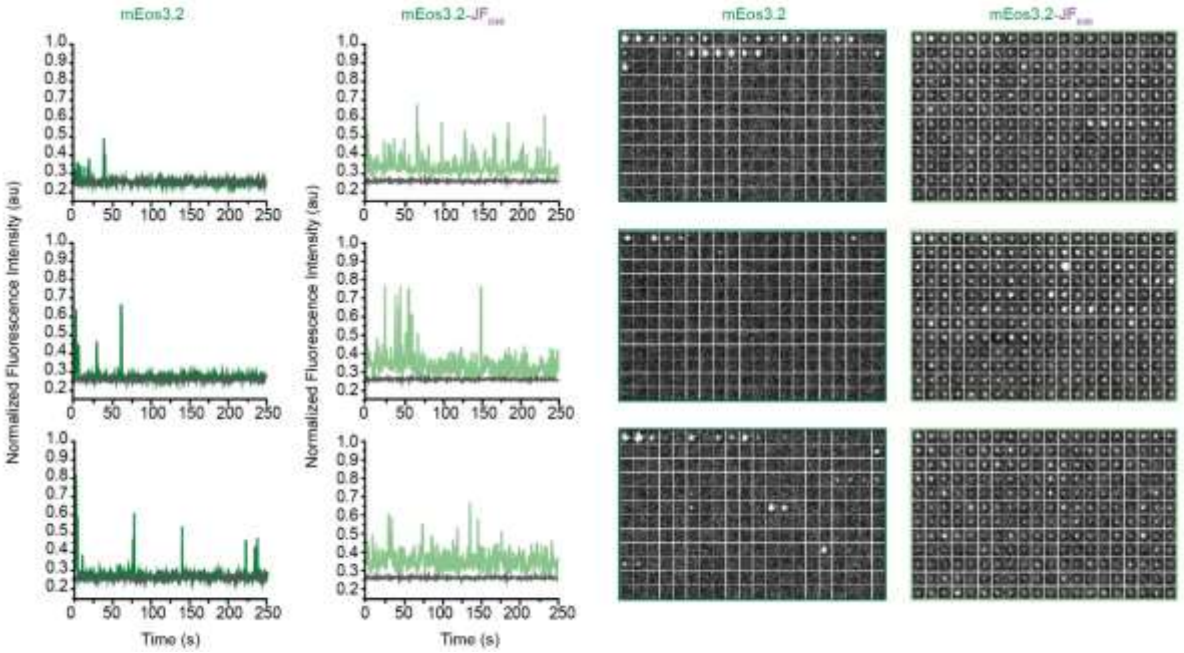

Supplementary Figure 3 | Heterogeneity in FRET efficiency observed in mEos3.2-JF$_{646}$ single-molecule traces. Example single-molecule traces of mEos3.2 emission for individual mEos3.2 (dark green) and mEos3.2-JF$_{646}$ molecules (light green) are shown with the surrounding background plotted separately (in grey). The generally lower intensity of the mEos3.2-JF$_{646}$ molecules is evident and they can be seen to occasionally increase intensity to a similar level to that of the mEos3.2 molecules – presumably as the JF$_{646}$ transiently switches to a triplet dark state before being quenched by the Trolox.

		Average precision (nm)
mEos3.2 CHD4	Figure 3	14.8 ± 0.9
mEos3.2-JF_{646} CHD4	Figure 3	28 ± 2
mEos3.2 CENP-A	Figure 4	15.0 ± 0.2
mEos3.2 CENP-A/JF_{646} CENP-A	Figure 4	20 ± 4
PA-JF_{549} H2B	Figure 5	11.9 ± 0.4
PA-JF_{549} H2B/JF_{646} H2B	Figure 5	13.3 ± 0.03

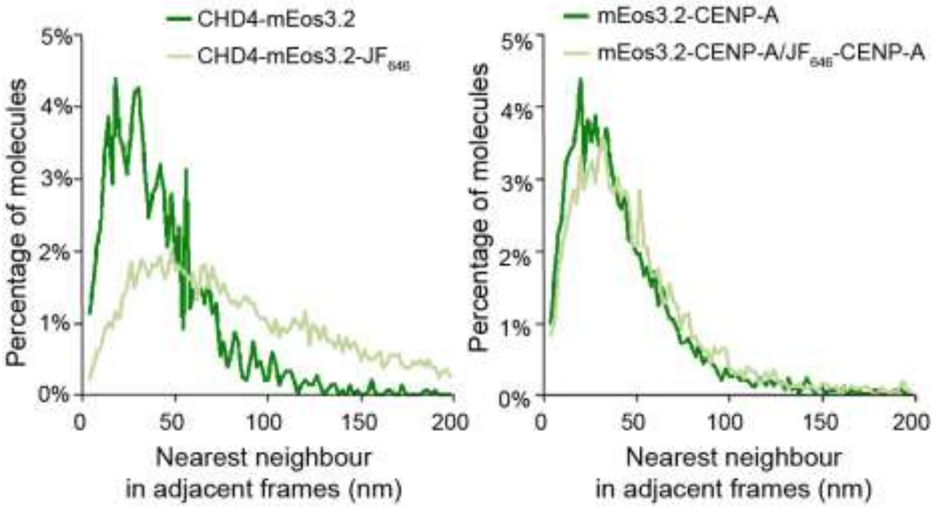

Supplementary Figure 4 | Localization precisions calculated for single-molecule imaging in mammalian cells. Average precisions calculated as previously described[1] are shown in the Table. The plots below show fits of the displacement *p*(d) to the nearest neighbor distribution of localizations in adjacent frames (corrected to account for contributions of different molecules to nearest neighbors), to yield experimental precisions for mEos3.2 and mEos3.2-JF_{646}.

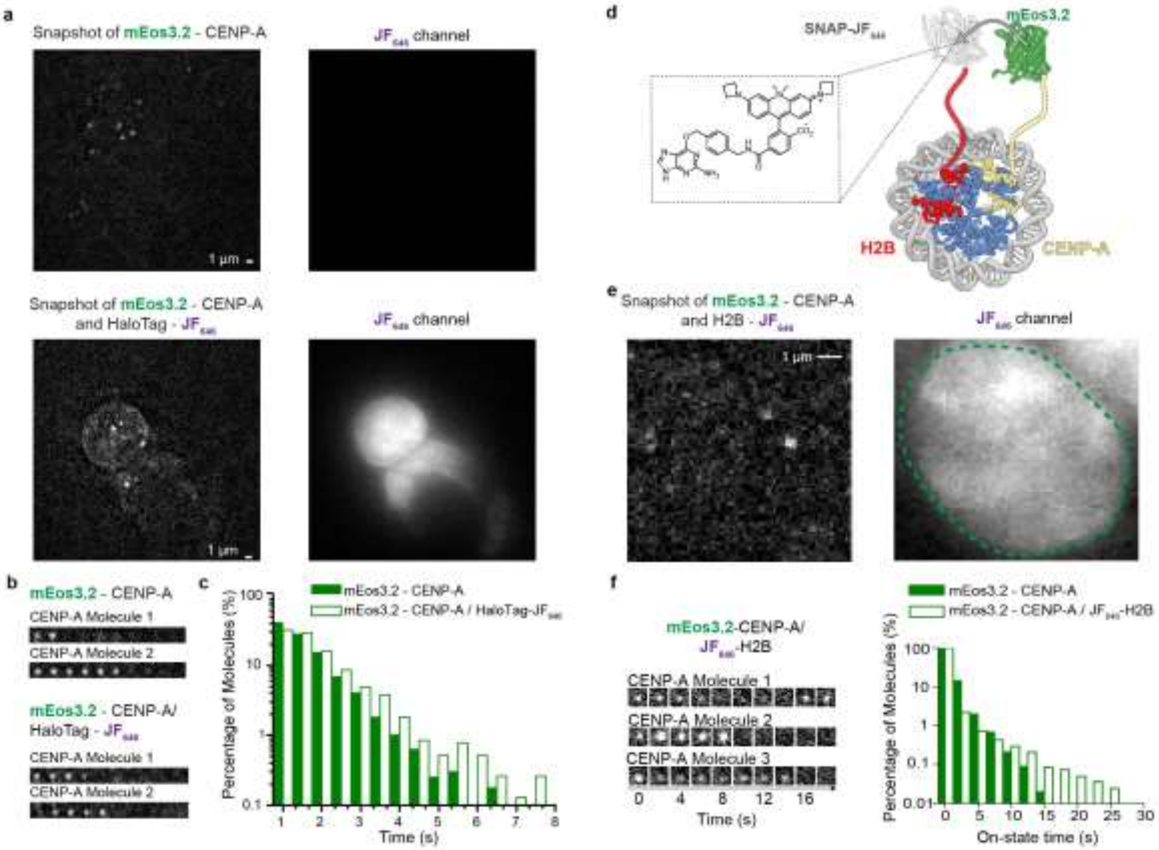

Supplementary Figure 5. mEos3.2-JF$_{646}$ allows tracking of single CENP-A protein complexes. a) (left) Representative 500 ms exposure images from the middle of the nucleus in mouse ES cells expressing mEos3.2-tagged CENP-A either alone or in the presence of freely diffusing JF$_{646}$-tagged HaloTag protein. (right) Images of the JF$_{646}$ dye show that the HaloTag has been successfully labeled. **b)** Montages of three representative 500 ms exposure single-molecule traces (one image for every 4 frames) are shown in the absence or presence of the JF$_{646}$-labeled HaloTag. **c)** Histograms showing the percentage of molecules remaining with a particular 'on-state' time (*i.e.* individual track length) after photo-conversion when performing sptPALM. Cells expressing mEos3.2-tagged CENP-A alone are compared with cells also expressing JF$_{646}$-tagged HaloTag. **d)** CENP-A molecules were fused with mEos3.2, and histone H2B with the SNAP tag to which JF$_{646}$ was coupled. (FRET can occur from mEos3.2-tagged CENP-A to the JF$_{646}$ dye on either the proximal or distal histone H2B molecule.) **e)** (left) Representative reconstruction, with an expansion of one the centromeres (inset), of localized single mEos3.2-tagged CENP-A molecules at centromeres are shown in the presence of JF$_{646}$-labeled histone H2B. (right) Images confirm that the JF$_{646}$ dye has successfully labeled SNAP-tagged histone H2B. **f)** Single mEos3.2-tagged CENP-A molecules show decreased intensity and increased track length in the presence of JF$_{646}$-tagged histone H2B. Montages of three representative 500 ms exposure single-molecule traces (showing an image for every 4 frames) are shown in the presence of JF$_{646}$-labeled histone H2B. Histograms showing the percentage of molecules with a particular 'on-state' time (i.e. individual track length) remaining after photo-conversion when performing sptPALM of

mouse ES cells. Cells expressing mEos3.2-tagged CENP-A are compared with cells also expressing JF_{646}-tagged histone H2B.

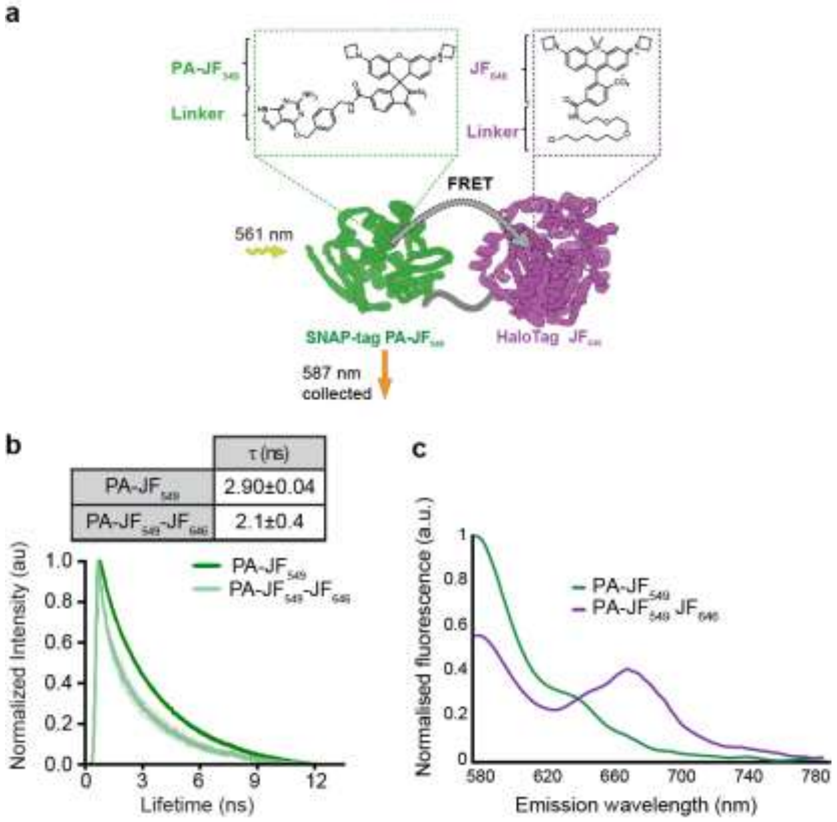

Supplementary Figure 6 | PA-JF$_{549}$-JF$_{646}$ shows FRET on the ensemble level. a) The fusion protein used as a test system to tether the JF$_{646}$ dye (via a HaloTag protein) next to PA-JF$_{549}$ (tethered to a SNAP tag protein). **b)** Fluorescence lifetime decay curves (background corrected and normalized to the maximum photon counts of each trace). The measured lifetimes (mean ± standard deviation, see inset) showed a decrease in fluorescence lifetime for PA-JF$_{549}$ in the presence of the JF$_{646}$ dye. **c)** Bulk emission spectra of PA-JF$_{549}$ in the absence and presence of JF$_{646}$ confirming the presence of FRET through a decrease in donor and increase in acceptor emission intensity. The spectra were produced as described in **Supplementary Fig. 2c**.

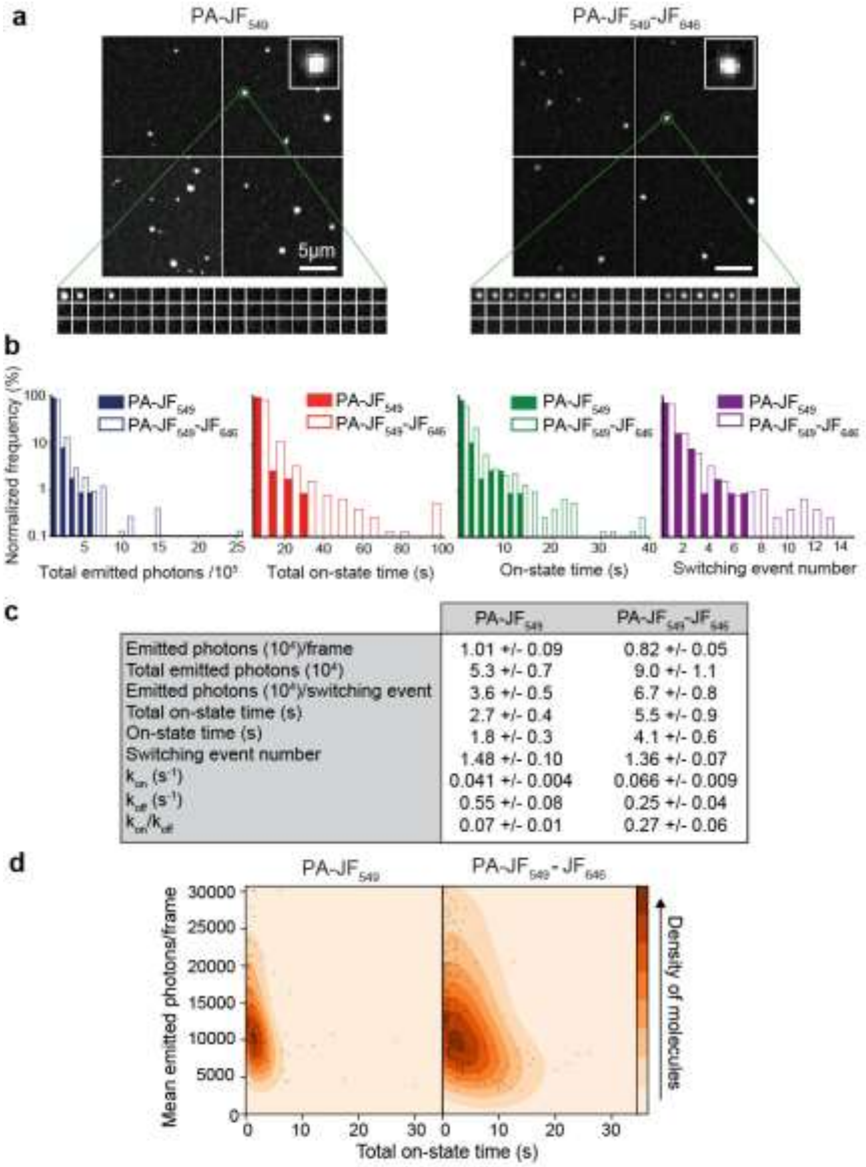

	PA-JF$_{549}$	PA-JF$_{549}$-JF$_{646}$
Emitted photons (10^4)/frame	1.01 +/- 0.09	0.82 +/- 0.05
Total emitted photons (10^4)	5.3 +/- 0.7	9.0 +/- 1.1
Emitted photons (10^4)/switching event	3.6 +/- 0.5	6.7 +/- 0.8
Total on-state time (s)	2.7 +/- 0.4	5.5 +/- 0.9
On-state time (s)	1.8 +/- 0.3	4.1 +/- 0.6
Switching event number	1.48 +/- 0.10	1.36 +/- 0.07
k_{on} (s^{-1})	0.041 +/- 0.004	0.066 +/- 0.009
k_{off} (s^{-1})	0.55 +/- 0.08	0.25 +/- 0.04
k_{on}/k_{off}	0.07 +/- 0.01	0.27 +/- 0.06

Supplementary Figure 7 | Single-molecule characterization of the PA-JF$_{549}$-JF$_{646}$ FRET pair shows an improvement in photophysical properties. a) Representative single-molecule traces of PA-JF$_{549}$ at the 587 nm donor emission (depicted here as a montage of frames over time from left to right and top to bottom) showed reduced intensity and a longer 'on-time' in the presence of the JF$_{646}$ dye. **b)** Histograms of total emitted photons, total 'on-state' time, 'on-state' time and total number of switching events per molecule, when performing single-molecule TIRF imaging using either PA-JF$_{549}$ (filled bars) or the PA-JF$_{549}$ -JF$_{646}$ FRET pair (open bars) under identical imaging conditions. Parameters were extracted as described in **Fig. 2a** and the **Methods. c)** Table of extracted parameters given as the mean and standard error of the mean. **d)** Total 'on-state' time and average intensity of single molecules when performing single-molecule TIRF imaging using either PA-JF$_{549}$ or the PA-JF$_{549}$-JF$_{646}$ FRET pair under identical imaging conditions.

[NB – The *in vitro* characterization experiments of PA-JF_{549} and PA-JF_{549}-JF_{646} were limited by the tendency of the purified SNAP-tag-HaloTag construct to form multimers, at least in part due to the presence of an exposed cysteine residue in the SNAP-tag protein. To combat this dithiothreitol (1mM DTT) was added and only dilute samples could be imaged. In addition, problems with surface adherence of the protein construct were experienced. The construct had a very weak affinity for positively charged poly-L-lysine and was prone to detach from this coated surface. The protein had a greater affinity for argon plasma cleaned glass, which was negatively charged, but still had a propensity to detach from the surface. Despite this improvement, washing procedures employed to reduce background by removing unbound molecules resulted in relatively few single-molecules present in the first frame per acquisition. To ensure that the results of this experiment were statistically relevant, approximately >20 acquisitions were collected for each condition. As a result error values for the extracted photophysical parameters could not be obtained from the standard deviation in the mean value of each acquisition and bootstrapping was used to determine the standard error of the mean.]

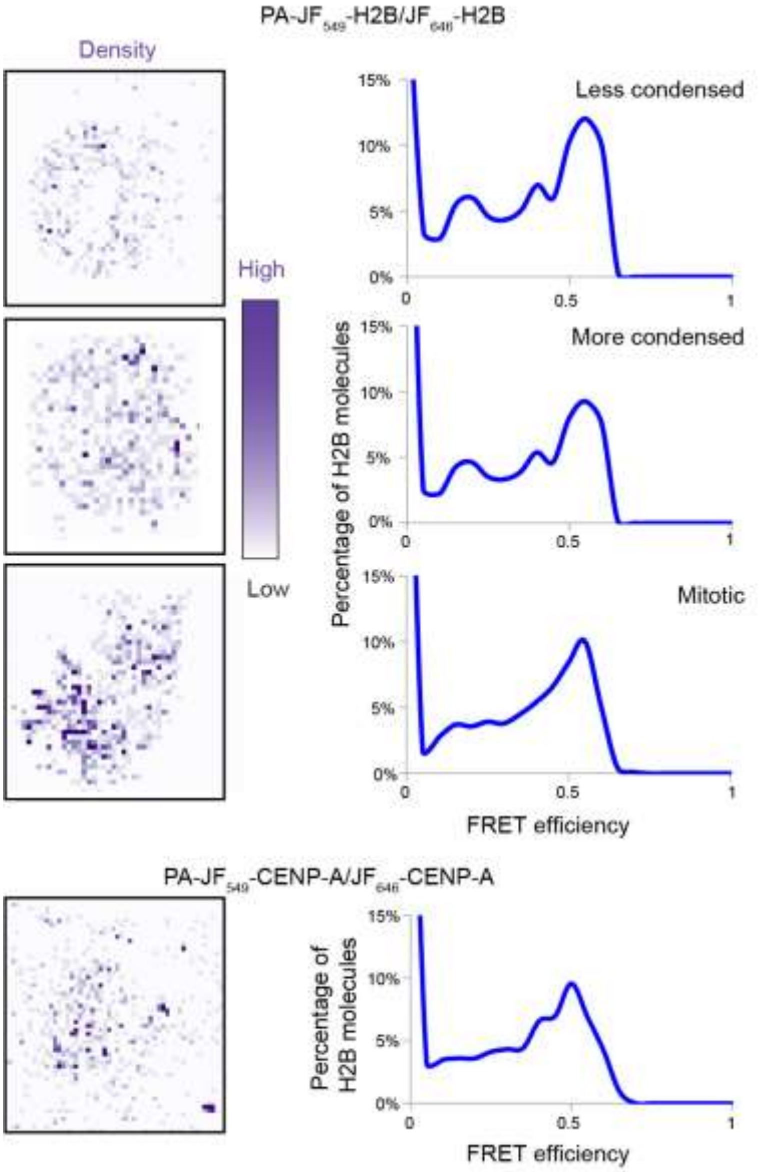

Supplementary Figure 8 | Several FRET states are observed for PA-JF$_{549}$-tagged histone H2B molecules in the presence of JF$_{646}$-tagged H2B. FRET efficiency was calculated from the intensity of individual trajectories of PA-JF$_{549}$-tagged H2B molecules in the presence of JF$_{646}$-tagged H2B. To determine the intensity expected for PA-JF$_{549}$ in the absence of FRET, we used the average intensity of PA-JF$_{549}$-tagged H2B molecules in the absence of JF$_{646}$-tagged H2B. The density of localized PA-JF$_{549}$ tagged H2B molecules is shown in purple for the three cells shown to the left, with less condensed, more condensed or mitotic chromatin. The distributions of molecules at varying FRET efficiency are shown to the right of each cell.

SUPPLEMENTARY REFERENCE

1 Endesfelder, U., Malkusch, S., Fricke, F. & Heilemann, M. A simple method to estimate the average localization precision of a single-molecule localization microscopy experiment. *Histochem Cell Biol* **141**, 629-638, doi:10.1007/s00418-014-1192-3 (2014).
