# Supplementary material for: FRET-enhanced photostability allows improved single-molecule tracking of proteins and protein complexes in live mammalian cells
Source: Nat Commun. 2018 Jun 28;9:2520. doi: 10.1038/s41467-018-04486-0 (PMC6023872; doi:10.1038/s41467-018-04486-0)
Supplement: Supplementary file 3 — Description of Additional Supplementary Files [file 41467_2018_4486_MOESM3_ESM.pdf]

Description of Additional Supplementary Files

File Name: Supplementary Movie 1
Description: TIRF imaging of single mEos3.2 molecules affixed to a coverslip and imaged (500 ms time resolution, 50 frames per second).

File Name: Supplementary Movie 2
Description: TIRF imaging of single mEos3.2-JF_{646} molecules affixed to a coverslip and imaged (500 ms time resolution, 50 frames per second).

File Name: Supplementary Movie 3
Description: Oblique-angle illumination imaging of single mEos3.2-JF_{646}-tagged CHD4 molecules in mouse embryonic stem cells (500 ms time resolution, 10 frames per second).

File Name: Supplementary Movie 4
Description: Oblique-angle illumination imaging of single mEos3.2-tagged CENP-A molecules in mouse embryonic stem cells (500 ms time resolution, 10 frames per second).

File Name: Supplementary Movie 5
Description: Oblique-angle illumination imaging of single mEos3.2-tagged CENP-A molecules in the presence of JF_{646}-tagged CENP-A molecules in mouse embryonic stem cells (500 ms time resolution, 10 frames per second).
